# Consumer Identification of Processed Foods and Their Health Effects

**DOI:** 10.1001/jamanetworkopen.2025.19518

**Published:** 2025-07-08

**Authors:** Neal D. Barnard, Anna Herby, Stephanie McBurnett, Hana Kahleova

**Affiliations:** 1 School of Medicine & Health Sciences, George Washington University, Washington, DC; 2 Physicians Committee for Responsible Medicine, Washington, DC

## Abstract

This survey study assesses public understanding of processed foods and their association with health in a representative sample of the US population.

## Introduction

To improve health, some have recommended avoiding ultraprocessed foods.^1^ However, in epidemiological studies, while processed meat consumption is associated with increased risk of diabetes and cardiovascular disease, many plant-based ultraprocessed foods (eg, breakfast cereals) are associated with reduced risk of these conditions.^2,3,4^ To assess public understanding of processed foods and their association with health, a survey of a representative US population sample was conducted.

## Methods

This survey study followed the American Association for Public Opinion Research (AAPOR) reporting guideline. The Advarra institutional review board waived approval because only deidentified information was collected. Data were collected from December 13 to 15, 2024, via a self-administered questionnaire. The online survey asked participants to identify processed foods, state whether they believed all processed foods to be unhealthy, and specify which foods increased type 2 diabetes risk (eAppendix in Supplement 1). Population data were weighted to approximate a target sample of US adults based on educational attainment, age, race, and region. Simple statistics were calculated for the overall sample and demographic subgroups based on race, ethnicity, and age. Proportions of participants giving a particular response were statistically compared within subgroups using a standard 2-tailed χ^2^ test. Statistical significance was set at *P* < .05.

## Results

This study included 2174 participants aged 18 to 92 years (unweighted No. [%], 643 aged 28 to 43 years [30%]; 1146 females [53%]; 227 Hispanic [10%]; 370 non-Hispanic Black [17%]; 1429 non-Hispanic White [66%]) (Table). No single processed food was identified by a respondent majority. Among the 2174 unweighted respondents, 609 (28%) cited meat products (eg, lunch meat, hot dogs, and hamburgers), 307 (14%) cited shelf-stable foods (eg, canned, packaged, frozen), and 289 (13%) cited foods with artificial additives (Figure). Half as many young respondents aged 18 to 27 years cited meat products (unweighted, 47 of 263 [18%]) compared with older respondents aged 60 to 92 years (unweighted, 261 of 727 [36%]; *P* < .001). The second most frequently identified foods among young participants were those with artificial additives, followed by chips or crackers, which were mentioned more frequently (unweighted, 34 of 263 [13%]) compared with older participants (unweighted, 43 of 727 [6%]; *P* < .001).

**Table.** Participant Characteristics

Characteristic	Participant, No. (%) (n = 2174)	
	Weighted	Unweighted
Age, y		
18-27	287 (13)	263 (12)
28-43	665 (31)	643 (30)
44-59	540 (25)	541 (25)
60-92	682 (31)	727 (33)
Sex		
Female	1112 (51)	1146 (53)
Male	1060 (49)	1025 (47)
Other or no answer	2 (0)	3 (0)
Ethnicity^a^		
Hispanic	380 (17)	227 (10)
Non-Hispanic	1794 (83)	1947 (90)
Race^a^		
American Indian	27 (1)	43 (2)
Asian	145 (7)	74 (3)
Black total	281 (13)	397 (18)
Non-Hispanic Black	263 (12)	370 (17)
White total	1670 (77)	1567 (72)
Non-Hispanic White	1334 (61)	1429 (66)
Other^b^	51 (2)	93 (4)
Education		
High school or less	830 (38)	677 (31)
Some college	572 (26)	852 (39)
College graduate	771 (35)	645 (30)

^a^ To determine whether responses differed based on race or ethnicity, participants were asked, “Are you, yourself, of Hispanic origin or descent, such as Mexican, Puerto Rican, or Cuban, or some other Spanish background?” and provided an opportunity to respond “yes” or “no.” They were then asked, “Which term below best describes your race or background?” Possible responses included “American Indian,” “Asian American,” “Black,” “White,” and “Other.” Population data were weighted to approximate a target sample of adults based on educational attainment, age, race, and region.

^b^ Other includes Native Hawaiian or Other Pacific Islander, more than 1 race, or did not specify.

**Figure.**
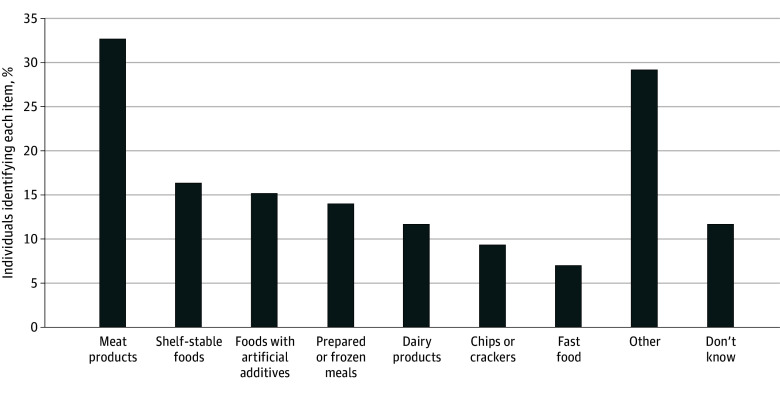
Items Identified as Processed Foods Among All 2174 Participants The data tabulate responses to the statement “Please give an example of what you would consider a processed food.” The other category includes (1) 3% of each of the following categories: sweets, such as cookies or cake; factory-made or manipulated foods; cereal; unhealthy food or junk food in general; and misinterpretations of the question; (2) 2% of each of the following categories: foods high in sugar or corn syrup; wheat products such as bread, pasta; pizza; and (3) 1% of each of the following categories: foods high in salt; foods high in fats and oils; and the statement everything is processed.

Among the 2174 weighted respondents, 840 (39%) reported believing that all processed foods were unhealthy (unweighted, 813 [37%]), and 875 (40%) did not believe all processed foods were unhealthy (unweighted, 883 [41%]). The remainder were unsure or did not know. Additionally, 82 of 145 weighted Asian respondents (57%) (unweighted, 41 of 74 [55%]; *P* < .001) and 166 of 380 weighted Hispanic respondents (44%) (unweighted, 104 of 227 [46%]; *P* = .003) were more likely than others to endorse the statement that all processed foods were unhealthy.

When asked which foods increased type 2 diabetes risk, 1101 unweighted respondents cited sugar (51%), 421 cited desserts (eg, candies, sweets, cookies, cakes) (19%), and 326 cited carbohydrates in general (15%). Additionally, 161 unweighted respondents (7%) mentioned processed foods, and only 17 mentioned meat products (1%).

## Discussion

Responses were highly subjective and largely unrelated to prior epidemiological research findings on the health effects of processed foods. Similarly, in a 2023 survey, healthiness of foods was strongly and inversely correlated with perceived processing levels (*r* = −0.96; *P* < .01).^5^ Most young participants failed to identify processed meat as a processed food. Although consumption of both processed and unprocessed meat is associated with diabetes risk, as well as colorectal cancer and cardiovascular risk, most young participants did not identify these products as increasing the risk of developing type 2 diabetes, a worrisome finding, given the rising incidence of diabetes in this group.^6^ Study limitations include the limited sample size and the fact that respondents to an online survey may not be representative of the larger population. The vague term *processed foods* should be replaced by more specific terms describing foods’ known health effects.

## Notes

Supplement 1. **eAppendix. **Survey Questions

Supplement 2.
Data Sharing Statement

## References

[1] Monteiro CA, Cannon G, Levy RB, . Ultra-processed foods: what they are and how to identify them. Public Health Nutr. 2019;22(5):936-941. doi:10.1017/S1368980018003762 30744710 PMC10260459

[2] Chen Z, Khandpur N, Desjardins C, . Ultra-processed food consumption and risk of type 2 diabetes: three large prospective US cohort studies. Diabetes Care. 2023;46(7):1335-1344. doi:10.2337/dc22-1993 36854188 PMC10300524

[3] Dicken SJ, Dahm CC, Ibsen DB, . Food consumption by degree of food processing and risk of type 2 diabetes mellitus: a prospective cohort analysis of the European Prospective Investigation into Cancer and Nutrition (EPIC). Lancet Reg Health Eur. 2024;46:101043. doi:10.1016/j.lanepe.2024.101043 39529810 PMC11551512

[4] Mendoza K, Smith-Warner SA, Rossato SL, . Ultra-processed foods and cardiovascular disease: analysis of three large US prospective cohorts and a systematic review and meta-analysis of prospective cohort studies. Lancet Reg Health Am. 2024;37:100859. doi:10.1016/j.lana.2024.100859 39286398 PMC11403639

[5] Hässig A, Hartmann C, Sanchez-Siles L, Siegrist M. Perceived degree of food processing as a cue for perceived healthiness: the NOVA system mirrors consumers’ perceptions. Food Qual Prefer. 2023;110:103944. doi:10.1016/j.foodqual.2023.104944

[6] Tönnies T, Brinks R, Isom S, . Projections of type 1 and type 2 diabetes burden in the US population aged <20 years through 2060: the SEARCH for DIABETES in Youth Study. Diabetes Care. 2023;46(2):313-320. doi:10.2337/dc22-0945 36580405 PMC9887625

