# Supplementary material for: Consumer Identification of Processed Foods and Their Health Effects
Source: JAMA Netw Open. 2025 Jul 8;8(7):e2519518. doi: 10.1001/jamanetworkopen.2025.19518 (PMC12238891; doi:10.1001/jamanetworkopen.2025.19518)
Supplement: Supplement 1. — eAppendix. Survey Questions [file jamanetwopen-e2519518-s001.pdf]

Supplemental Online Content

Barnard ND, Herby A, McBurnett S, Kahleova H. Consumer identification of processed foods and their health effects. *JAMA Network Open*. 2025;8(7):e2519518.
doi:10.1001/jamanetworkopen.2025.19518

eAppendix. Survey Questions

This supplemental material has been provided by the authors to give readers additional information about their work.

© 2025 Barnard ND et al. *JAMA Network Open*.

eAppendix. Survey Questions

The survey included the following statements/questions:

1. Scientists are studying the effects of processed foods on health. Please give an example of what you would consider a processed food. (Multiple responses were permitted.)

2. Do you believe all processed foods are unhealthy?

3. Now, please think about the condition type 2 diabetes. To the best of your knowledge, which foods increase the risk of a person developing type 2 diabetes?

© 2025 Barnard ND et al. *JAMA Network Open*.
