# Supplementary material for: Consumer Identification of Processed Foods and Their Health Effects
Source: JAMA Netw Open. 2025 Jul 8;8(7):e2519518. doi: 10.1001/jamanetworkopen.2025.19518 (PMC12238891; doi:10.1001/jamanetworkopen.2025.19518)
Supplement: Supplement 2. — Data Sharing Statement [file jamanetwopen-e2519518-s002.pdf]

**Data Sharing Statement**

**Data**
**Data available:** Yes
**Data types:** Deidentified participant data
**How to access data:** nbarnard@pcrm.org
**When available:** With publication

**Supporting Documents**
**Document types:** None

**Additional Information**
**Who can access the data:** Anyone requesting the data
**Types of analyses:** For any purpose
**Mechanisms of data availability:** The data will be shared without a signed data agreement.
**Any additional restrictions:** None
